# Epidemiology of Group A rotavirus in rodents and shrews in Bangladesh

**DOI:** 10.1007/s11259-022-09923-6

**Published:** 2022-04-05

**Authors:** Ariful Islam, Mohammad Enayet Hossain, Ausraful Islam, Shariful Islam, Md. Kaisar Rahman, Rashedul Hasan, Mojnu Miah, Mohammed Ziaur Rahman

**Affiliations:** 1 EcoHealth Alliance, New York, NY 10018 USA; 2 Centre for Integrative Ecology, School of Life and Environmental Science, Deakin University, Victoria, Australia; 3 Institute of Epidemiology, Disease Control and Research (IEDCR), Dhaka, Bangladesh; 4 International Centre for Diarrheal Diseases Research, Bangladesh (icddr,b), Dhaka, Bangladesh

**Keywords:** Prevalence, Bandicota, *Mus musculus*, *Rattus rattus*, *Suncus murinus*, Rotavirus

## Abstract

Rodents and shrews live in close proximity to humans and have been identified as important hosts of zoonotic pathogens. This study aimed to detect Group A rotavirus (RVA) and its potential risk factors in rodents and shrews in Bangladesh. We captured 417 small mammals from 10 districts with a high degree of contact between people and domestic animals and collected rectal swab samples between June 2011 and October 2013. We tested the swab samples for RVA RNA, targeting the NSP3 gene segment using real-time reverse transcription-polymerase chain reaction (rRT-PCR). Overall, RVA prevalence was the same (6.7%) in both rodents and shrews. We detected RVA RNA in 5.3% of *Bandicota bengalensis* (4/76; 95% CI: 1.4–12.9), 5.1% of *B. indica* (4/79; 95% CI: 1.4–12.4), 18.2% of *Mus musculus* (4/22; 95% CI: 5.2–40.3), 6.7% of *Rattus rattus* (6/90; 95% CI: 2.5–13.9), and 6.7% of *Suncus murinus* (10/150; 95% CI: 3.2–11.9). We found significantly more RVA in males (10.4%; OR: 3.4; *P* = 0.007), animals with a poor body condition score (13.9%; OR: 2.7; *P* = 0.05), during wet season (8.3%; OR: 4.1; *P* = 0.032), and in urban land gradients (10.04%; OR: 2.9; *P* = 0.056). These findings form a basis for understanding the prevalence of rotaviruses circulating among rodents and shrews in this region. We recommend additional molecular studies to ascertain the genotype and zoonotic potential of RVA circulating in rodents and shrews in Bangladesh.

## Introduction

Group A rotavirus (RVA) causes acute dehydrating diarrhea (Wenman et al. 1979; Parashar et al. 2006) in humans, especially children, and animals worldwide. Annual 128,500 deaths and 258,173,300 cases of diarrhea in children <5 years of age are attributable to RVA infection (Troeger et al. 2018). RVA is commonly transmitted via the fecal and oral routes (Anderson and Weber 2004). However, water, food, fomites (de Wit et al. 2003), and flies (*Musca domestica*) (Tan et al. 1997) can also be a source of infection for humans. Common symptoms of RVA include vomiting and diarrhea in children but nausea, malaise, headache, abdominal cramping, diarrhea, and fever in adults (Anderson and Weber 2004).

Different animal species, including small mammals, are infected with RVA (Dhama et al. 2009). Rodents and shrews belong to a diverse group of small mammals and are found widely throughout the world (Meerburg et al. 2009). They often live in close proximity to humans and domestic animals. Small mammals have the highest capacity for successful adaptation. Twenty-two species of rodents are found in Bangladesh (Khan 2013). Among them, *M. musculus* (house mouse), *R. rattus* (black rat/house rat), *Bandicota indica* (greater bandicoot rat), and *B. bengalensis* (Indian mole rat) are very common (Islam et al. 2020). *M. musculus* and *R. rattus* are listed among the 100 of the world’s most invasive alien species (Lowe et al. 2000). Of the 160 million people in Bangladesh, 13.6 million are exposed to rodents every month, and 8.5% (95%CI: 7.9–9.1) of people have direct contact with rodents (Shanta et al. 2016).

Small mammals’ species richness, density, and diversity are an indicator of a healthy and stable ecosystem (Avenant 2003). Although rodents and shrews play an essential role in ecology, they act as reservoirs of zoonotic pathogens (Meerburg et al. 2009) like Hantavirus (Wang et al. 2000; Radosa et al. 2013), *Yersinia pestis*, Rickettsia (typhus), Leptospira (Weil’s disease), Toxoplasma, Trichinella, Hepatitis E (Favorov et al. 2000), Bartonella (Ellis et al. 1999), Borrelia, Babesia, Anaplasma and Ehrlichia (Tadin et al. 2016). Rodents can transmit more than 20 diseases to humans through blood-sucking parasites (fleas, ticks, and mites) (Singla et al. 2008). Rodents and shrews are usually found near human and animal habitations at high densities as well as in other habitats like woodland and abandoned warehouses (Veciana et al. 2012). Due to urbanization and deforestation, wild small mammal populations come to human localities. Human-animal contact frequently occurs, which boosts the chance of cross-species transfer of zoonoses, including rotavirus (Sumangali et al. 2007), and poses a health risk to humans (Paramasvaran et al. 2009). Currently, zoonotic rotavirus infection in humans is more frequent than in the past, and various studies have reported the interspecies transmission of rotavirus from animals to humans (Doro et al. 2015; Martella et al. 2010). There is no way to rule out the possibility of rotaviruses becoming more pathogenic and increasing their transmissibility to humans through reassortment with other genotypes of RVA, similar to influenza viruses (Cowley et al. 2013; Li et al. 2016). Small mammals pose a significant risk to human health, especially those that have direct or indirect contact with animals. However, viruses carried by small mammals have not been well investigated in Bangladesh. The evolution and emergence of rotaviruses in the context of human health is an enigma, and it is public health to better understand the diversity, evolution, and origins of rotaviruses in small mammals.

In Bangladesh, one study reported a 64% prevalence of rotavirus among hospitalized children 5 years of age and younger admitted with acute gastroenteritis (Satter et al. 2017). There is an opportunity for cross-species transmission of zoonotic pathogens, including rotaviruses, because of the higher percentage of human cases, the high density of the human population, frequent contact with animals, including small mammals, and lack of awareness regarding hygiene and sanitation.

Data on rotavirus in small mammals are limited in the Indian subcontinent. RVA was isolated from urban wild rats (*R. norvegicus*) in Germany (Sachsenröder et al. 2014) and Brazil (Tonietti et al. 2013). Common shrews (*Sorex araneus*) (21.7%) in Germany were found to be infected with RVA (Johne et al. 2019). The prevalence of RVA in different rodents and shrew species in China was found to be 1.06% and 2.67%, respectively, by RT-PCR (Li et al. 2016), whereas rotaviral antigen was detected in 6.7% of samples from wild tree shrews (*Tupaia belangeri* Chinese) using ELISA (Wang et al. 2011). Due to the segmented nature of the viral genome, animal RVA can easily infect and adapt to humans through reassortment with other RVAs (Ianiro et al. 2017). Similarly, diverse strains of human RVA have been found that share genetic and antigenic features with animal RVA strains (Ianiro et al. 2017). The G3 genotype frequently infects humans, pigs, dogs and cats, horses, bats, and murine rodents (Geletu et al. 2021; Simsek et al. 2021). Murine RVA strains prolifically infect and multiply in mice only. Even though cross-species transmission of RVA from mice to humans has never been documented, animal-associated RVA is prevalent. However, within the last few years, interspecies transmission and genetic assortment between human and animal rotaviruses from cows, pigs, cats, and dogs have been reported (He et al. 2017; Sawant et al. 2020). Hence, it can be speculated that the cross-species transmission of RVA might occur between humans and rodents in Bangladesh. However, there is no published literature on RVA in rodents and shrews in Bangladesh to our knowledge. Therefore, the study aimed to determine the prevalence of RVA in the rodent and shrew population of Bangladesh. This will help conduct further research on their zoonotic potential in the future.

## Methods

### Study sites and duration

**Fig. 1:**
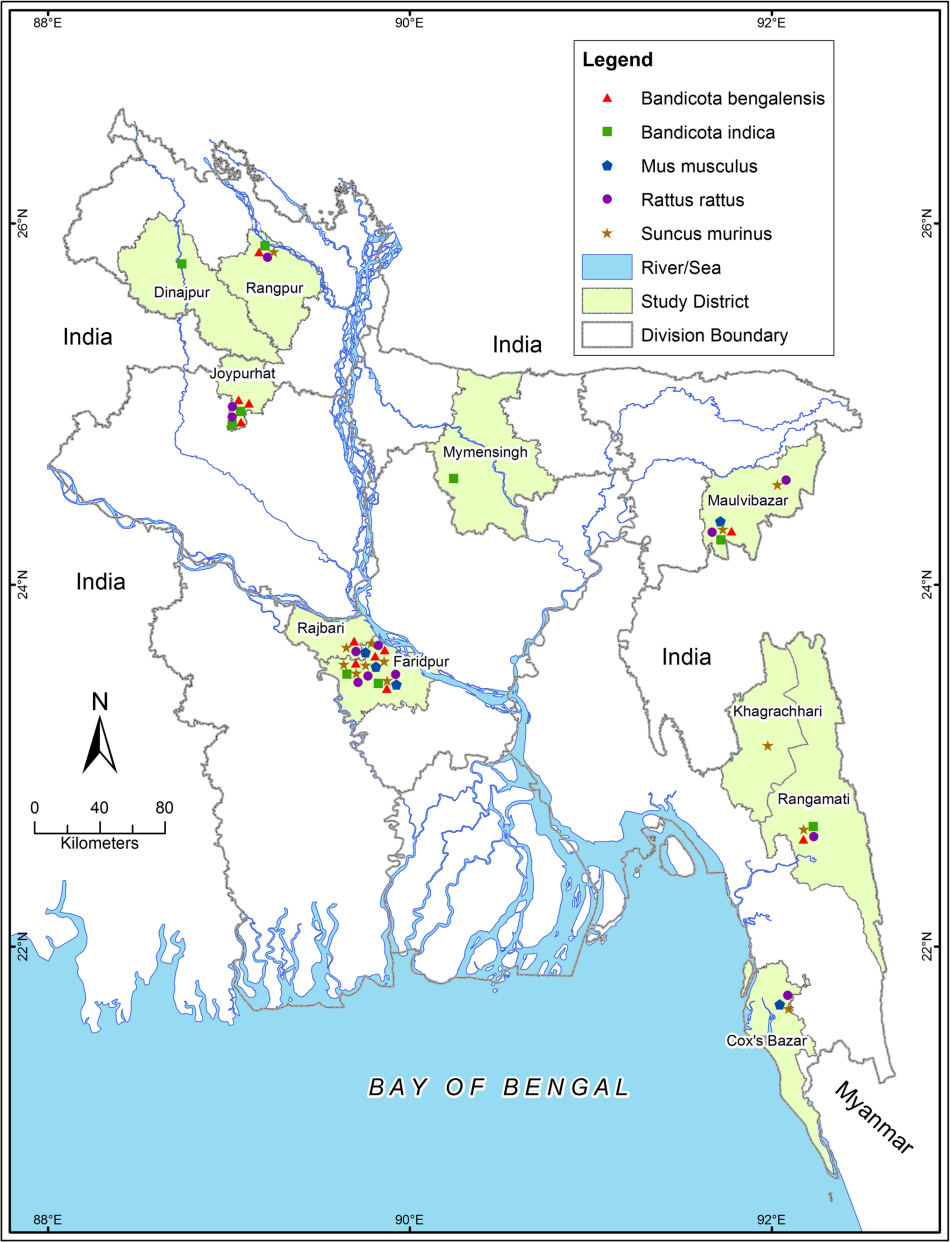
A map of Bangladesh displaying the sampling locations and spatial distribution of rodent and shrew species captured in the study areas from June 2011 to October 2013. The map was plotted using the spatial analyst tool of ArcGIS (ArcMap, version 10.2, Environmental Systems Research Institute, CA, USA) (Available at https://www.arcgis.com/index.html). Bangladesh’s administrative and study site shapefiles were retrieved from Humanitarian Data Exchange v1/1.43.6, the United Nations Office for the Coordination of Humanitarian Affairs (Available at https://data.humdata.org). Red triangles depict *B. bengalensis*, green squares indicate *B. indica*, blue pentagonsdepict *M. musculus*, purple circles denote *Rattus rattus,* and yellow star dot depicts *Suncus murinus*

From June 2011 to October 2013, we captured rodents and shrews from 10 different districts (Faridpur, Mymensingh, Rajbari, Rangamati, Khagrachhari, Dinajpur, Maulvibazar, Rangpur, Cox’s Bazar, and Joypurhat) in Bangladesh (Fig. 1). We selected sampling sites that were high-risk interfaces where frequent animal-human contact occurs. We categorized the selected study sites based on different land gradients as i) urban areas having a high population density and built environment infrastructure; and ii) Rural areas having a comparatively low population density with more agricultural land and less infrastructure. We captured the rodents and shrews from human dwellings, agricultural fields, and bushland in both urban and rural areas.

### Animal capture, sample collection, and data recording

We captured live rodents and shrews using locally made steel wire traps (27 cm × 13 cm × 13 cm) that have proven efficacy in sampling medium- and large-sized small mammals. We baited the traps with ghee-smeared biscuits and dried fish. We set the traps in human dwellings, agricultural fields, or bushland at dusk after obtaining verbal permission from the owners and collected them at dawn the next day. As rodents and shrews are considered pests, there was no objection from the owners of the properties, and we got their full consent and cooperation. We anesthetized the trapped animals using isoflurane following the procedures described elsewhere (Shafiyyah et al. 2012; Rahman et al. 2018). We collected rectal swabs and/or feces from each captured animal. Our research protocol and methods were carried out in accordance with the national institutional ethics committee of the International Centre for Diarrheal Disease Research, Bangladesh (icddr,b) (reference number: 2008–074) and the international ethics committee of the University of California, Davis Institutional Animal Care and Use Committee (IACUC #16048). After sampling, we released all the animals at the sites of capture. We identified the species of small mammals based on their morphological characteristics as described by Aplin et al. (Aplin et al. 2003). We recorded the lengths of the head, body, tail, hindfoot, and ear and the bodyweight; these parameters helped determine the species and age classes of the animals.

We captured a total of 417 small mammals consisting of *B. Indica* (*N* = 79), *B. bengaleensis* (*N* = 76), *M. musculus* (*N* = 22), *R. rattus* (*N* = 90), and *S. murinus* (*N* = 150). We placed the swab samples into 0.5 mL lysis buffer (NucliSENS Lysis Buffer, BIOMERIEUX, France) in a 1.8 mL cryotube (Corning, USA). We then stored the cryotubes in a liquid nitrogen dewar (Princeton Cryogenics, NJ, USA) immediately after collection in the field until their transfer to a -80 °C freezer at the icddr,b laboratory. We used a data sheet to record information like location, habitat, gradient of sampling sites, season and prominent anthropogenic changes, species, age, sex, morphometric measurements, Body Condition Score (BCS), and health status. We categorized the age groups as i) juvenile: weaned, independent from parental nursing, and without developed secondary sexual characteristics like descended testicles in males and ii) adults: matured in size and weight, with developed secondary sexual characteristics (Rahman et al. 2019). We categorized the land gradient as urban or residential, rural, and agricultural land (crop or pastureland). During data collection, we classified the BCS according to Hickman and Swan (2010), as emaciated (BCS-1), under conditioned (BCS-2), well-conditioned (BCS-3), over-conditioned (BCS-4), and obese (BCS-5). Finally, we regrouped the BCS-1 and BCS-2 as poor, BCS-3 as fair, and BCS-4 and BCS-5 as good.

### RNA extraction, and PCR

According to the manufacturer’s instructions, we extracted viral RNA from 200 μL rectal swab samples using the magnetic particle-based InviMag Virus DNA/RNA Mini kit (STRATEC Molecular GmbH, Germany); the final elution volume was 100 μL. We tested rectal swabs for RVA RNA by rRT-PCR using NSP3-specific primers and probes using the AgPath-ID One-Step RT-PCR system (Ambion Inc. Austin, USA) (Table 1) as described by Jothikumar et al. (2009) and Islam et al. (2020a). We performed conventional RT-PCR using the QIAGEN® One-Step RT-PCR kit (QIAGEN, Germany) to amplify the VP7 and VP4 gene fragments using consensus primer pairs Beg9/End9 and Con2/Con3 for identifying the G and P genotype, respectively (Table 1), as described by Rahman et al. (2007) and Islam et al. (2020b). We used MOCK (only lysis) and known RVA positive samples during the extraction process to ensure proper nucleic acid extraction.

**Table 1:** Oligonucleotide primers used in the study for PCR amplification

Primer	Target segment of RVA genome	Position	Strand	Sequence (5′−3′)	References
JVKF	NSP3	17–39	Plus	CAGTGGTTGATGCTCAAGATGGA	Jothikumar et al. (2009)
JVKR	NSP3	147–123	Minus	TCATTGTAATCATATTGAATACCCA	Jothikumar et al. (2009)
JVKP	NSP3	96–72	Plus	FAM-ACAACTGCAGCTTCAAAAGAAGWGT-BHQ1	Jothikumar et al. (2009)
Beg9	VP7	1–28	Plus	GGCTTTAAAAGAGAGAATTTCCGTCTGG	Gouvea et al. (1990)
End9	VP7	1062–1036	Minus	GGTCACATCATACAATTCTAATCTAAG	Gouvea et al. (1990)
Con2	VP4	868–887	Minus	ATTTCGGACCATTTATAACC	Gentsch et al. (1992)
Con3	VP4	11–32	Plus	TGGCTTCGCCATTTTATAGACA	Gentsch et al. (1992)

### Statistical analysis

We entered the data into MS Excel-2013 (Microsoft office excel-2013, USA) and imported it to STATA-13 (StataCorp, 4905, Lakeway Drive, College Station, Texas 77,845, USA) for analysis. We performed descriptive statistics for different variables using Fisher’s exact test. Then, we forwarded the variables (p < 0.2) to multivariable logistic regression and checked for confounding. We tested the model’s goodness of fit using the Hosmer–Lemeshow test. Additionally, the predictive ability of the model was determined using the Receiver Operating Characteristic (ROC) curve (Dohoo et al. 2003). The area under the curve (AUC) was categorized as acceptable (AUC = 0.7 to 0.8), excellent (AUC = 0.8–0.9), and outstanding (AUC = 0.9 to 1.0) (Dw 2000; Sayeed et al. 2017). We considered differences among the variables to be significant if p < 0.05.

## Results

We detected a 6.7% (*n* = 28) prevalence of RVA in the sampled small mammals but could not amplify any G or P type among the RVA-positive samples. We found similar percentages of RVA in both rodents (*n* = 18; 6.7%; 95%CI: 4.04–10.45) and shrews (*n* = 10; 6.7%; 95%CI: 3.2–11.9). REgarding animal species, *M. musculus* (*n* = 4; 18.2%; 95%CI: 5.2–40.3) had the highest and *B. indica* (*n* = 4; 5.1%; 95%CI: 1.3–12.5) the lowest percentage of RVA. We did not find any significant variation in RVA prevalence between juveniles (*n* = 3; 6.1%; 95% CI: 1.3–16.9) and adult (*n* = 25; 6.8%; 95%CI: 4.44–9.9). Significantly more male animals (*n* = 20; 10.4%; 95%CI: 6.5–15.6) were infected with RVA than females (*n* = 8; 3.6%; 95%CI: 1.6–6.9; *p* = 0.006). Animals from urban area (*n* = 23; 10.04%; 95%CI: 6.4–14.7) were more likely to be positive for RVA than animals from rural areas (*n* = 5; 2.66%; 95%CI: 0.87–6.1) (*p* = 0.003). We observed that significantly less rodents and shrews from agricultural fields or bushlands were infected than those from human dwellings (*p* = 0.039) (Table 2).

**Table 2:** The association of selected variables and RVA presence in small mammals (*N* = 417) from Bangladesh

Variables	Category	N	RVA-positive *n* (%)	*p* value* (Fisher’s exact)
Types of mammals	Rodent	267	18 (6.7)	1.00
	Shrew	150	10 (6.7)	
Species	*Bandicota bengalensis*	76	4 (5.3)	0.325
	*B. indica*	79	4 (5.1)	
	*Mus musculus*	22	4 (18.2)	
	*Rattus rattus*	90	6 (6.7)	
	*Suncus murinus*	150	10 (6.7)	
Age	Adult	368	25 (6.8)	1.000
	Juvenile	49	3 (6.1)	
Sex	Female	225	8 (3.6)	0.006
	Male	192	20 (10.4)	
Land gradient	Urban	229	23 (10.04)	0.003
	Rural	188	5 (2.66)	
Habitat type	Agricultural field/bushland	150	5 (3.3)	0.042
	Human dwelling	267	23 (8.6)	
BCS	Poor	72	10 (13.9)	0.016
	Good	345	18 (5.2)	
Health status	Apparently healthy	394	24 (6.1)	0.059
	Sick	23	4 (17.4)	
Season	Dry	115	3 (2.6)	0.047
	Wet	302	25 (8.3)	

*The p values are for unconditional significance tests for differences among categories within each variable

Multivariable logistic regression analysis revealed that males had 3.4 times higher risk of having RVA than females (95% CI: 1.39–8.04; *p* = 0.007), whereas animals with poor BCS were 2.7 times more susceptible to RVA than those with a good BCS (95% CI: 0.99–7.38; *p* = 0.05). Samples collected during the wet season were 4.1 times more likely to be positive than those obtained during the dry season (*p* = 0.032). Additionally, small mammals from urban areas had a 2.9 times higher risk of being RVA-positive than animals from rural areas (95% CI: 0.97–9.15; *p* = 0.056) (Table 3).

**Table 3:** Multivariable logistic regression model for identifying key risk factors for RVA in rodents and shrews in Bangladesh

Variables	Factors	Odds ratio	95% CI*		*p* value
			Lower bound	Upper bound	
Sex	Female	1.0			
	Male	3.4	1.39	8.04	0.007
BCS	Good	1.0			
	Poor	2.7	0.99	7.38	0.05
Health status	Apparently healthy	1.0			
	Sick	0.8	0.19	3.48	0.789
Season	Dry	1.0			
	Wet	4.1	1.13	14.65	0.032
Land gradient	Rural	1.0			
	urban	2.9	0.97	9.15	0.056
Habitat type	Agricultural field/bushlands	1.0			
	Human dwelling	1.8	0.59	5.79	0.284

*Confidence interval

**Fig. 2:**
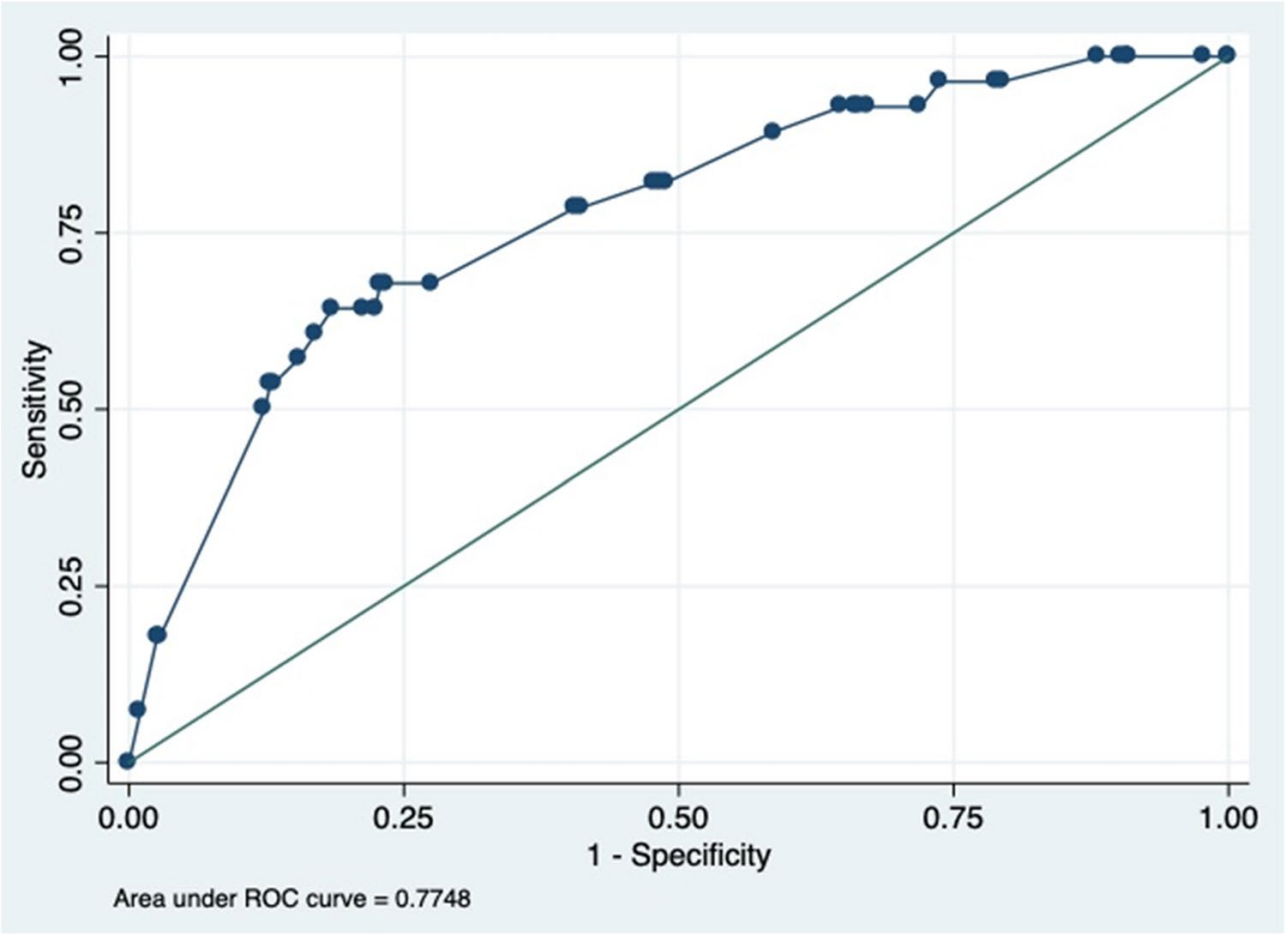
The plot of sensitivity versus 1-specificity for a receiver operating characteristic curve (ROC) of various parameters of the logistic model of RVA in small mammals in Bangladesh

We validated the model using the ROC curve (area under curve, AUC 77%) (Fig. 2). The plot of sensitivity versus 1-specificity depicts the predictability of the logistic model across various parameters associated with RVA in small mammals of Bangladesh.

## Discussion

The study detected RVA in different species of rodents and shrews in several areas in Bangladesh. Previous studies identified RVA in other species of rodents and shrews using RT-PCR in Germany (Sachsenröder et al. 2014), China (Li et al. 2016), Brazil (Tonietti et al. 2013), and New York (Williams et al. 2018). In contrast, studies in Australia (McInnes et al. 2011), West Indies (Boey et al. 2019), and Western Europe (Mähler and Köhl 2009) reported rotavirus antibodies in rodents. However, published literature on RVA in small mammals is scarce worldwide. To the authors’ knowledge, the present study is the first to report RVA in rodents and shrews in different habitat types and land gradients in Bangladesh. Previous studies reported P and G genotypes of RVA infection in humans and animals in Bangladesh, strongly suggesting the possibility of zoonotic transmissions (Dey et al. 2020; Mazid et al. 2020; Sharif et al. 2020). Thus, the current study findings extend the known host range of RVA in Bangladesh and will be helpful for the rest of the world prevent and control the spillover of RVA from rodents and shrews.

We detected RVA in *Rattus rattus*, but in Brazil and Germany, RVA was seen in *R. norvegicus* (Tonietti et al. 2013; Sachsenröder et al. 2014). Similarly, we identified RVA in *M. musculus*, whereas studies in Australia reported it in *Mus musculus domesticus,* a subspecies of *M. musculus* (Singleton et al. 1993; Smith et al. 1993). However, our study did not genotype *R. rattus* and *Mus musculus* mice. Nevertheless, earlier studies reported the presence of both *R. norvegicus* and *M. musculus doemesticus* from Bangladesh (Adhikari et al. 2018; Barman et al. 2020). The prevalence of RVA in our studied rodents was higher than in previous studies (Li et al. 2016; Ianiro et al. 2017), but in the case of shrews, our results are similar to those of a former study from China that found a 6.7% seroprevalence in wild tree shrews (*Tupaia belangeri* Chinese) using antigen-capture ELISA (Wang et al. 2011). A study in Germany reported a 21.7% prevalence of RVA in common shrews, which is much higher than in our study (Johne et al. 2019). The higher prevalence may be due to the differences in host species (*Sorex araneus*) and the wide circulation of RVA in shrews from different regions in Germany.

A comparatively high prevalence of RVA was found in rodents and shrews in urban areas. Rodent and shrew density is higher in urban and peri-urban areas compared to rural areas in Bangladesh (Shanta et al. 2016). Moreover, more than 90% of urban and peri-urban household members observed rodents and shrews on their premises, where 8.5% of respondents reported direct contact with them. A previous study from Bangladesh reported a high density of rodents and shrews in urban areas, making them more susceptible to infectious agents either by functional suppression of the immune system caused by a viral infection, malnutrition, or the stress induced by overcrowding (Smith et al. 1993). Besides, urban areas are densely populated with humans where rodents and shrews are considered pests. These small mammals live in human houses (Veciana et al. 2012). They collect food at night and pick up contaminated food from human sources, which may also expose them to RVA. Moreover, RVA is circulating in domestic and wild animals in developing countries like Bangladesh, where humans and animals live in proximity and have frequent interactions (Hossain et al. 2020). This also indicates the possible transmission of RVA from humans to small mammals (Ianiro et al. 2017), which may explain the greater positivity rate of samples near human dwellings.

We found the highest odds ratio for RVA presence in *M. musculus* but could not find any previous study to compare our results. However, some studies have evaluated the presence of antibodies against RVA in different animals. One study estimated the seroprevalence of RVA in laboratory mice and rats in Western Europe and found the highest seroprevalence in mice (Mähler and Köhl 2009). We found RVA in a higher percentage of small mammals sampled during the wet season. A study conducted on *M. musculus domesticus* by Singleton et al. (1993) also found similar trends of seroprevalence during the wet season (April to September) in Southern Australia. The authors also reported increased mouse densities over time. Though we did not record any animal density-related information, this may be the case in our study.

Additionally, serology does not confirm the presence of infection, instead indicating recent infection. All the positive samples from sick animals were collected during the wet season. From April to September (wet season), comparatively little food is available for the animals. Moreover, the quality of food available is not adequate, resulting in malnutrition among small mammals. September to October is also the breeding season, creating social stress on male mice (Singleton et al. 1993). All these factors may influence viral prevalence and persistence in the studied animals.

Sex and BCS also influenced the presence of RVA in our study, but the reason behind this is not clear. One explanation may be that male mammals are more active than females, which predisposes them to infection from various sources. Sometimes, BCS provides more precise information to assess the health status of animals (Hickman and Swan 2010). Usually, animals with poor body conditions are immune-compromised and have limited access to food, which makes them susceptible to different infectious diseases (Smith et al. 1993). However, we admit that the observed relationship between health conditions and RVA presence in the animals may be due to a sampling artifact. We captured a disproportionately higher percentage of rodents and shrews in the wet seasons, corresponding to a study in Uganda (Ssuuna et al. 2020). Environmental factors influence the composition and abundance of rodent species, and rapid growth of vegetation occurs during the rainy season, providing shelter and food for rodents and shrews. Besides, human activities differ concerning different months and seasons, influencing the capture of small mammals (Mulungu et al. 2003).

Human infections associated with group A, B, and C rotavirus are common in Bangladesh (Dey et al. 2020), but the rodent-borne RVA is unknown as data regarding the incidence of zoonotic human RVA infections in Bangladesh are not available. We found RVA in rodents and shrews in Bangladesh, with some critical factors related to RVA infection in peri-domestic species. However, it is crucial to establish longitudinal surveillance across multiple regional animal populations to detect and genetically characterize the RVA. This study did not focus on proving human infection from rodents and shrews. However, as rodents and shrews often live in close contact with humans, although the presence of RVA in rodents and shrews in this study was low (6.7%), it would not be rational to overlook the risk of small mammal borne RVA infection in humans. On the other hand, human RVA strains cannot infect mice under laboratory conditions. Therefore, it is also unlikely that human RVA will infect rodents and shrews (Ciarlet et al. 2002).

The study has some limitations. The primers and probes used were not designed for rodents. However, these primers and probes have a wide range and are used for detecting RVA in clinical and environmental samples. We detected RVA in macaques, bats, and domestic animals using the same primers targeting the NSP3 gene in earlier studies (Hossain et al. 2020; Islam et al. 2020a; Islam et al. 2020b). In the current study, we did not have access to rodent-specific internal positive controls (IPC). Using IPCs would have excluded the possibility of getting false-negative results. We also could not successfully amplify and sequence the VP4 and VP7 genes from RVA-positive samples. This may be due to lower nucleic acid content in the swab samples.

Additionally, we cannot ignore the genetic diversity of RVA in rodents, which may impact the performance of the primers used in this study (Čolić et al. 2021). Nevertheless, next-generation sequencing and/or primer-independent approaches are needed to improve the characterization of RVAs in the fecal samples of rodents and shrews in Bangladesh. We recommend establishing longitudinal surveillance to detect and genetically characterize RVA among multiple host populations, conduct DNA barcoding to identify spatial patterns in these species.

## Conclusions

Taken together, the findings of this study suggest that RVA is circulating in rodents and shrews in Bangladesh. Similar to other wildlife species, it is difficult to detect and sequence RVA in small mammals. As rodents and shrews often live in close contact with humans as well as with farm and pet animals, they form a distinct and significant nexus between wildlife communities and human populations. We recommend further studies on the molecular characterization of RVA in rodents and shrews, their epizootiology, and possible risks to humans at different land gradients in Bangladesh.

## Data Availability

All datasets used and/or analyzed during this study are included in this article and are available from the corresponding author on reasonable request.

## References

[CR1] Adhikari P, Han SH, Kim YK, Kim TW, Thapa TB, Subedi N, Adhikari P, Oh HS (2018). First molecular evidence of Mus musculus bactrianus in Nepal inferred from the mitochondrial DNA cytochrome B gene sequences. Mitochondrial DNA Part A.

[CR2] Anderson EJ, Weber SG (2004). Rotavirus infection in adults. Lancet Infect Dis.

[CR3] Aplin KP, Brown PR, Jacob J, Krebs CJ, Singleton GR (2003) Field methods for rodent studies in Asia and the indo-Pacific. ACIAR Monograph No

[CR4] Avenant NL (2003) The use of small mammal community characteristics as an indicator of ecological disturbance in the Korannaberg conservancy. ACIAR Monograph Series 96:95–98. https://citeseerx.ist.psu.edu/viewdoc/download?doi=10.1.1.470.6426&rep=rep1&type=pdf#page=92. Accessed 20 Dec 2020

[CR5] Barman A, Abdullah SM, Ali Y, Rahman M, Mohanta UK (2020). Prevalence and detail morphological identification of helminths of murine rodents in Dhaka city, Bangladesh. Ann Parasitol.

[CR6] Boey K, Shiokawa K, Avsaroglu H, Rajeev S (2019). Seroprevalence of rodent pathogens in wild rats from the island of St. Kitts, West Indies. Animals.

[CR7] Ciarlet M, Conner ME, Finegold MJ, Estes MK (2002). Group A rotavirus infection and age-dependent diarrheal disease in rats: a new animal model to study the pathophysiology of rotavirus infection. J Virol.

[CR8] Čolić D, Krešić N, Mihaljević Ž, Andreanszky T, Balić D, Lolić M, Brnić D (2021). A remarkable genetic diversity of rotavirus A circulating in red fox population in Croatia. Pathogens.

[CR9] Cowley D, Donato CM, Roczo-Farkas S, Kirkwood CD (2013). Novel G10P [14] rotavirus strain, northern territory, Australia. Emerg Infect Dis.

[CR10] de Wit MA, Koopmans MP, van Duynhoven YT (2003). Risk factors for norovirus, Sapporo-like virus, and Group A rotavirus gastroenteritis. Emerg Infect Dis.

[CR11] Dey SK, Sharif N, Sarkar OS, Sarkar MK, Talukder AA, Phan T, Ushijima H (2020). Molecular epidemiology and surveillance of circulating rotavirus among children with gastroenteritis in Bangladesh during 2014–2019. PLoS One.

[CR12] Dhama K, Chauhan R, Mahendran M, Malik S (2009). Rotavirus diarrhea in bovines and other domestic animals. Vet Res Commun.

[CR13] Dohoo IR, Martin W, Stryhn HE (2003). Veterinary epidemiologic research.

[CR14] Doro R, Farkas SL, Martella V, Bányai K (2015). Zoonotic transmission of rotavirus: surveillance and control. Expert Rev Anti-Infect Ther.

[CR15] Dw L (2000). Applied logistic regression analysis.

[CR16] Ellis B, Regnery R, Beati L, Bacellar F, Rood M, Glass G, Marston E, Ksiazek T, Jones D, Childs J (1999). Rats of the genus Rattus are reservoir hosts for pathogenic Bartonella species: an Old World origin for a New World disease?. J Infect Dis.

[CR17] Favorov MO, Kosoy MY, Tsarev SA, Childs JE, Margolis HS (2000). Prevalence of antibody to hepatitis E virus among rodents in the United States. J Infect Dis.

[CR18] Geletu US, Usmael MA, Bari FD (2021). Rotavirus in calves and its zoonotic importance. Vet Med Intern.

[CR19] Gentsch JR, Glass RI, Woods P, Gouvea V, Gorziglia M, Flores J, Das BK, Bhan MK (1992). Identifi cation of Group A rotavirus gene 4 types by polymerase chain reaction. J Clin Microbiol.

[CR20] Gouvea V, Glass RI, Woods P, Taniguchi K, Clark HF, Forrester B (1990). Polymerase chain reaction amplifi cation and typing of rotavirus nucleic acid from stool specimens. J Clin Microbiol.

[CR21] He B, Huang X, Zhang F, Tan W, Matthijnssens J, Qin S, Xu L, Zhao Z, Yang LE, Wang Q (2017) Group A rotaviruses in Chinese bats: genetic composition, serology, and evidence for bat-to-human transmission and reassortment. J Virol 91(12):e02493-16. 10.1128/jvi.02493-16 10.1128/JVI.02493-16 PMC5446661 28381569

[CR22] Hickman DL, Swan M (2010). Use of a body condition score technique to assess health status in a rat model of polycystic kidney disease. J Am Assoc Lab Anim Sci.

[CR23] Hossain MB, Rahman MS, Watson OJ, Islam A, Rahman S, Hasan R, Kafi MAH, Osmani MG, Epstein JH, Daszak P (2020). Epidemiology and genotypes of Group A rotaviruses in cattle and goats of Bangladesh, 2009-2010. Infect Genet Evol.

[CR24] Ianiro G, Di Bartolo I, De Sabato L, Pampiglione G, Ruggeri FM, Ostanello F (2017). Detection of uncommon G3P [3] rotavirus A (RVA) strain in rat possessing a human RVA-like VP6 and a novel NSP2 genotype. Infect Genet Evol.

[CR25] Islam S, Rahman MK, Ferdous J, Rahman M, Akter S, Faraque MO, MNU C, Hossain MA, Hassan MM, Islam A, Islam A (2020). Hemoprotozoa and Anaplasma spp. in rodents and shrews of Bangladesh. Trop Biomed.

[CR26] Islam A, Hossain ME, Haider N, Rostal MK, Mukharjee SK, Ferdous J, Miah M, Rahman M, Daszak P, Rahman MZ (2020). Molecular characterization of Group A rotavirus from rhesus macaques (Macaca mulatta) at human–wildlife interfaces in Bangladesh. Transbound Emerg Dis.

[CR27] Islam A, Hossain ME, Rostal MK, Ferdous J, Islam A, Hasan R, Miah M, Rahman M, Rahman MZ, Daszak P (2020). Epidemiology and molecular characterization of rotavirus A in fruit bats in Bangladesh. EcoHealth.

[CR28] Johne R, Tausch SH, Grützke J, Falkenhagen A, Patzina-Mehling C, Beer M, Höper D, Ulrich RG (2019). Distantly related rotaviruses in common shrews, Germany, 2004–2014. Emerg Infect Dis.

[CR29] Jothikumar N, Kang G, Hill V (2009). Broadly reactive TaqMan® assay for real-time RT-PCR detection of rotavirus in clinical and environmental samples. J Virol Methods.

[CR30] Khan R, Khan R (2013). Wildlife of the Sundarban. Sundarban rediscovering Sundarban the mangrove beauty of Bangladesh.

[CR31] Li K, Lin XD, Huang KY, Zhang B, Shi M, Guo WP, Wang MR, Wang W, Xing JG, Li MH (2016). Identification of novel and diverse rotaviruses in rodents and insectivores, and evidence of cross-species transmission into humans. Virol.

[CR32] Lowe S, Browne M, Boudjelas S, De Poorter M (2000) 100 of the world's worst invasive alien species: a selection from the global invasive species database, Invasive Species Specialist Group Auckland. https://portals.iucn.org/library/sites/library/files/documents/2000-126.pdf. Accessed 20 Dec 2020

[CR33] Mähler M, Köhl W (2009). A serological survey to evaluate contemporary prevalence of viral agents and mycoplasma pulmonis in laboratory mice and rats in western Europe. Lab animal.

[CR34] Martella V, Bányai K, Matthijnssens J, Buonavoglia C, Ciarlet M (2010). Zoonotic aspects of rotaviruses. Vet Microbiol.

[CR35] Mazid R, Aung M, Paul S, Ahmad F, Alam M, Ali M, Nath P, Ahmed S, Haque N, Hossain M (2020). Resurgence and predominance of G3P [8] human rotaviruses in north-Central Bangladesh, 2018–2019. New Microbes New Infect.

[CR36] McInnes EF, Rasmussen L, Fung P, Auld AM, Alvarez L, Lawrence DA, Quinn ME, Utteridge TD, Del Fierro GM, Vassallo BA (2011). Prevalence of viral, bacterial and parasitological diseases in rats and mice used in research environments in Australasia over a 5-y period. Lab Animal.

[CR37] Meerburg BG, Singleton GR, Kijlstra A (2009). Rodent-borne diseases and their risks for public health. Crit Rev Microbiol.

[CR38] Mulungu LS, Makundi RH, Leirs H, Massawe AW, Vibe-Petersen S, Stenseth NC (2003). The rodent density-damage function in maize fields at an early growth stage. ACIAR Monograph Series.

[CR39] Paramasvaran S, Sani RA, Hassan L, Kaur H, Krishnasamy M, Jeffery J, Raj S, Ghazali SM, Hock LK (2009). Endo-parasite fauna of rodents caught in five wet markets in Kuala Lumpur and its potential zoonotic implications. Trop Biomed.

[CR40] Parashar UD, Gibson CJ, Bresee JS, Glass RI (2006). Rotavirus and severe childhood diarrhea. Emerg Infect Dis.

[CR41] Radosa L, Schlegel M, Gebauer P, Ansorge H, Heroldová M, Jánová E, Stanko M, Mošanský L, Fričová J, Pejčoch M (2013). Detection of shrew-borne hantavirus in Eurasian pygmy shrew (*Sorex minutus*) in Central Europe. Infect Genet Evol.

[CR42] Rahman M, Sultana R, Ahmed G, Nahar S, Hassan ZM, Saiada F, Podder G, Faruque ASG, Siddique AK, Sack DA, Matthijnssens J, Ranst MV, Azim T (2007). Prevalence of G2P [4] and G12P [6] rotavirus, Bangladesh. Emerg Infect Dis.

[CR43] Rahman M, Islam S, Masuduzzaman M, Alam M, Chawdhury MNU, Ferdous J, Islam MN, Hassan MM, Hossain MA, Islam A (2018). Prevalence and diversity of gastrointestinal helminths in free-ranging Asian house shrew (*Suncus murinus*) in Bangladesh. Vet World.

[CR44] Rahman MK, Islam S, Rahman M, Ferdous J, Akter S, Rahaman MM, Hossain MA, Hassan MM, Islam A (2019). Hematological and biochemical reference values of Asian house shrews (*Suncus murinus*) in Bangladesh. Vet World.

[CR45] Sachsenröder J, Braun A, Machnowska P, Ng TFF, Deng X, Guenther S, Bernstein S, Ulrich RG, Delwart E, Johne R (2014). Metagenomic identification of novel enteric viruses in urban wild rats and genome characterization of a Group A rotavirus. J Gen Virol.

[CR46] Satter SM, Gastanaduy PA, Islam K, Rahman M, Rahman M, Luby SP, Heffelfinger JD, Parashar UD, Gurley ES (2017). Hospital-based surveillance for rotavirus gastroenteritis among young children in Bangladesh: defining the potential impact of a rotavirus vaccine program. Pediatr Infect Dis J.

[CR47] Sawant PM, Digraskar SS, Gopalkrishna V (2020). Molecular characterization of unusual G10P [33], G6P [14] genomic constellations of Group A rotavirus and evidence of zooanthroponosis in bovines. Infect Genet Evol.

[CR48] Sayeed MA, Smallwood C, Imam T, Mahmud R, Hasan RB, Hasan M, Anwer MS, Rashid MH, Hoque MA (2017). Assessment of hygienic conditions of live bird markets on avian influenza in Chittagong metro, Bangladesh. Prev Vet Med.

[CR49] Shafiyyah COS, Jamaiah I, Rohela M, Lau YL, Aminah FS (2012). Prevalence of intestinal and blood parasites among wild rats in Kuala Lumpur, Malaysia. Trop Biomed.

[CR50] Shanta I, Luby S, Hossain K, Ahmed S, Rahman T, Kennedy E, Sharker M, Kilpatrick A, Pulliam J, Gurley ES (2016). Determining hotspots of human exposure to rodents, bats and monkeys in Bangladesh. Int J Infect Dis.

[CR51] Sharif N, Nobel NU, Sakib N, Liza SM, Khan ST, Billah B, Parvez AK, Haque A, Talukder AA, Dey SK (2020). Molecular and epidemiologic analysis of diarrheal pathogens in children with acute gastroenteritis in Bangladesh during 2014–2019. Pediatr Infect Dis J.

[CR52] Simsek C, Corman VM, Everling HU, Lukashev AN, Rasche A, Maganga GD, Binger T, Jansen D, Beller L, Deboutte W, Gloza-Rausch F, Seebens-Hoyer A, Yordanov S, Sylverken A, Oppong S, Sarkodie YA, Vallo P, Leroy EM, Bourgare M, Yinda KC, Ranst MV, Drosten C, Drexler JF, Matthijnssens J (2021). At least seven distinct rotavirus genotype constellations in bats with evidence of reassortment and zoonotic transmissions. MBio.

[CR53] Singla LD, Singla N, Parshad VR, Juyal PD, Sood NK (2008). Rodents as reservoirs of parasites in India. Integr Zool.

[CR54] Singleton GR, Smith AL, Shellam G, Fitzgerald N, Müller WJ (1993). Prevalence of viral antibodies and helminths in field populations of house mice (Mus domesticus) in southeastern Australia. Epidemiol Infect.

[CR55] Smith AL, Singleton GR, Hansen GM, Shellam G (1993). A serologic survey for viruses and mycoplasma pulmonis among wild house mice (Mus domesticus) in southeastern Australia. J Wildl Dis.

[CR56] Ssuuna J, Makundi RH, Isabirye M, Sabuni CA, Babyesiza WS, Mulungu LS (2020). Rodent species composition, relative abundance, and habitat association in the Mabira central Forest reserve, Uganda. J Vertebr Biol.

[CR57] Sumangali K, Rajakaruna R, Rajapakse R (2007) Ecto and Endo parasites of rodents from two selected sites in Kandy District. Peradeniya university research sessions purse 2007 Volume 12 Part I-Agricultural, Biological Med Sci Edit Board 44(3&4):86. https://www.researchgate.net/profile/Sarath-Kodithuwakku/publication/242414576_Factors_Influencing_the_Student_Decision_Making_in_Relation_to_University_Admission/links/53ea933e0cf2fb1b9b6a6c03/Factors-Influencing-the-Student-Decision-Making-in-Relation-to-University-Admission.pdf. Accessed 20 Dec 2020

[CR58] Tadin A, Tokarz R, Markotić A, Margaletić J, Turk N, Habuš J, Svoboda P, Vucelja M, Desai A, Jain K (2016). Molecular survey of zoonotic agents in rodents and other small mammals in Croatia. Am J Trop Med Hyg.

[CR59] Tan S, Yap K, Lee HL (1997). Mechanical transport of rotavirus by the legs and wings of Musca domestica (Diptera: Muscidae). J Med Entomol.

[CR60] Tonietti PDO, da Hora AS, Silva FDF, Ferrari KL, Brandão PE, Richtzenhain LJ, Gregori F (2013) Simultaneous detection of Group A rotavirus in swine and rat on a pig farm in Brazil. Sci World J 2013:648406. 10.1155/2013/648406 10.1155/2013/648406 PMC3671536 23766702

[CR61] Troeger C, Khalil IA, Rao PC, Cao S, Blacker BF, Ahmed T, Armah G, Bines JE, Brewer TG, Colombara DV (2018). Rotavirus vaccination and the global burden of rotavirus diarrhea among children younger than 5 years. JAMA Pediatr.

[CR62] Veciana M, Chaisiri K, Morand S, Ribas A (2012) Helminths of the Asian house shrew *Suncus murinus* from Cambodia. Camdodian J Nat Hist 2012(2):115–122. https://www.academia.edu/3035160/Helminths_of_the_Asian_house_shrew_Suncus_murinus_from_Cambodia. Accessed 20 Dec 2020

[CR63] Wang H, Yoshimatsu K, Ebihara H, Ogino M, Araki K, Kariwa H, Wang Z, Luo Z, Li D, Hang C (2000). Genetic diversity of hantaviruses isolated in China and characterization of novel hantaviruses isolated from Niviventer confucianus and Rattus rattus. Virol.

[CR64] Wang XX, Li JX, Wang WG, Sun XM, He CY, Dai JJ (2011). Preliminary investigation of viruses to the wild tree shrews (*Tupaia belangeri chinese*). Zool Res.

[CR65] Wenman WM, Hinde D, Feltham S, Gurwith M (1979). Rota virus infection in adults. N Engl J Med.

[CR66] Williams SH, Che X, Garcia JA, Klena JD, Lee B, Muller D, Ulrich W, Corrigan RM, Nichol S, Jain K (2018) Viral diversity of house mice in New York City. MBio 9(2):e01354-17. 10.1128/mbio.01354-17 10.1128/mBio.01354-17 PMC5904411 29666290

